# Sequencing by Hybridization of Long Targets

**DOI:** 10.1371/journal.pone.0035819

**Published:** 2012-05-04

**Authors:** Yu Qin, Tobias M. Schneider, Michael P. Brenner

**Affiliations:** School of Engineering and Applied Sciences and Kavli Institute for Bionano Science and Technology, Harvard University, Cambridge, Massachusetts, United States of America; University of North Carolina at Charlotte, United States of America

## Abstract

Sequencing by Hybridization (SBH) reconstructs an *n*-long target DNA sequence from its biochemically determined *l*-long subsequences. In the standard approach, the length of a uniformly random sequence that can be unambiguously reconstructed is limited to 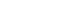 due to repetitive subsequences causing reconstruction degeneracies. We present a modified sequencing method that overcomes this limitation without the need for different types of biochemical assays and is robust to error.

## Introduction

Sequencing by Hybridization (SBH) [1]–[3] uses the binding characteristics of a library of short DNA probes (oligonucleotides) to reconstruct a target DNA sequence. Traditionally, SBH has been carried out using microarrays [4], but recent advances in microfluidics have created the possibility of carrying out the hybridization reactions inside of small droplets in high throughput [5], [6]. This creates new opportunities for using SBH creatively for sequencing. A fundamental limit to the length *n* of the target sequence that can be sequenced by probes of length l follows from an information theoretic bound: since there are 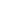 probes in a standard probe library, each of which may or may not bind to a subsequence of the target, the probe library can give 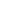 possible measurements; comparing this with the 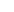 possible target sequences implies that for a unique measurement to be associated with every possible target sequence, we need 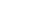, or 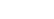. Hence, for probes of length 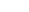, the maximum target sequence length is 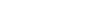.

In fact, the maximum length of the target that can be sequenced is much below the above threshold due to repetitive subsequences. Denote those oligonucleotides that bind to the target the *spectrum* of the target. If, for example, two oligonucleotides in the spectrum, *b* and *c*, have their 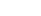-mer prefixes both identical to the 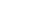-mer suffix of a third oligonucleotide *a*, then there are *two* possible target sequences, with either *b* or *c* as the successor of *a*, that are consistent with the probe binding characteristics. Analysis of uniformly random target sequences shows that this results in an SBH reconstruction boundary 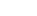 [7], [8], reducing the maximum length of a target that can be sequenced with probes of length 7 to 128. The dramatic decrease in the length of sequenceable targets has severely limited the efficacy of SBH [9]. An additional challenge is biochemical errors in measuring the probe spectrum. For moderate error rates, recent heuristic reconstruction algorithms [10]–[12] can however overcome both positive and negative errors and perform close to the intrinsic reconstruction boundary, i.e. 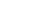 (for information on existing algorithms see the information S1). To increase the length of sequenceable targets while fixing the size of the probe library, theoretical concepts have previously revolved around using optimized probe patterns involving non-specifically binding universal bases instead of standard oligonucleotide probes [13], which have proven hard to implement.

In this paper, we present a methodology that overcomes the problem of degeneracy due to multiple repeats, is robust to errors and only requires the use of standard oligonucleotide probes. The idea is to use the fact that although repeats cause the probe binding characteristics to correspond to multiple possible target sequences, the set of possibilities for a target sequence can be completely enumerated. By randomly fragmenting multiple copies of the target sequence the resulting probe binding data of the fragments can be combined to uniquely identify the target sequence. Numerical simulations demonstrate that this sequencing method outperforms the classical 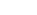 boundary for random sequences, has excellent performance on natural sequences, and is robust to error.

## Results

Reconstructing a target sequence from its subsequences in the presence of errors is a computationally complex problem [14], requiring the use of heuristic search algorithms which randomly find one of the possibly multiple solutions. We implement a reconstruction algorithm based on the ant colony optimization (ACO) [12], and demonstrate that the failure of the classical SBH method is indeed correlated with the structure of subsequence repeats.

**Figure 1:**
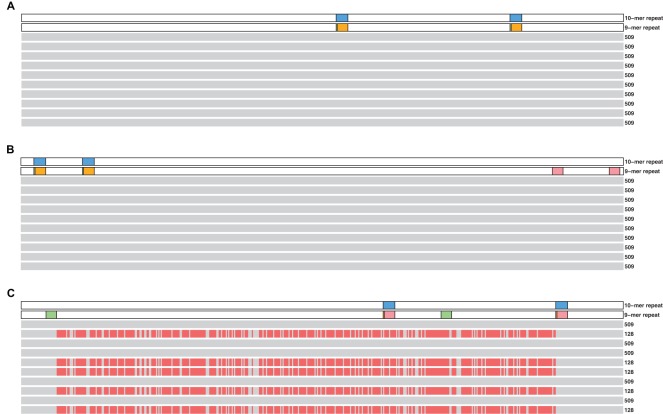
Sequencing failure due to degenerate solutions. Classical sequencing by hybridization of three representative 509-mer target sequences taken from a benchmark library [15] using 10-mer probes. For each target sequence, 10 independent reconstruction attempts using a state-of-the-art heuristic algorithm (ACO [12]) are simulated and the results are visualized with grey (pink) color representing bases that are correctly (incorrectly) matched by the reconstruction. Reconstruction failures are correlated with the locations of 9-mer but not with 10-mer repeats within the target sequence, shown in the upper two rows of each figure. For two interlaced 9-mer repeats (C), two different solutions with Needleman-Wunsch similarity score [17] between the target and the reconstruction of 509 and 128 are found. Both sequences have the same 10-mer probe spectrum and can thus not be distinguished using classical SBH. For reconstruction attempts of other target sequences see the online applet [16] and instructions in the information S1.

Using the 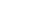-mer spectrum, we simulate the reconstruction of natural DNA sequences of length 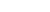, taken from a benchmark library [15], a library that contains 10-mer repeats. FIG. 1 shows 10 independent simulated reconstruction attempts of three representative instances of the benchmark library (Numbers 21, 3 and 20) with random 1% positive and 1% negative errors added to each spectrum. Visualizing the repeat structure of the target DNA demonstrates that the existence of multiple solutions with degenerate spectra requires at least two pairs of 9-mer repeats, i.e. repeats one base shorter than the probes, arranged in an appropriate configuration. As a result, the single pair of 9-mer repeats in FIG. 1A does not lead to any reconstruction errors, nor does the double pair of 9-mer repeats in FIG. 1B, where the second pair of repeats directly follows the first. In contrast, FIG. 1C shows that if the 9-mer repeats interlace each other, half of the reconstructions have significant reconstruction error of the same pattern right after a 9-mer repeat, but switches back to the correct solution exactly at the beginning of a second 9-mer repeat. This ‘wrong’ reconstruction is actually a second solution compatible with the same spectrum. These rules persist in our reconstructions of the entire benchmark library (see the online applet [16] and instructions in the information S1).

A symbolic representation further demonstrates that both the number of 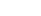-mer repeats and their aligning pattern are part and parcel for the non-uniqueness of solutions. Suppose we have a sequence with two pairs of 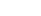-mer repeats, with subsequences denoted by 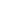 and 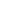, respectively. If the target sequence is 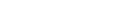, with 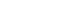 and *F* arbitrary (nonrepeating) subsequences, then another possible reconstruction of this sequence is 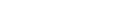. Note that this second reconstruction, which switches the subsequence 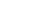 with 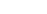, has exactly the same spectrum as the target sequence. In contrast, if two pairs of repeats are arranged in the target sequence as 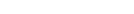, then there is no rearrangement of the subsequences that leads to a reconstructed sequence with the same spectrum as the target. In general, given repeats 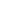 and 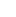, if there exist two subsequences of the target sequence of the form 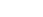 and 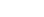, then interchanging these subsequences leads to a reconstruction with a consistent spectrum. In the case of a triple repeat, i.e. a target sequence of the form 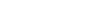, a second sequence which matches the spectrum is 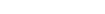.

## Methods

**Figure 2:**
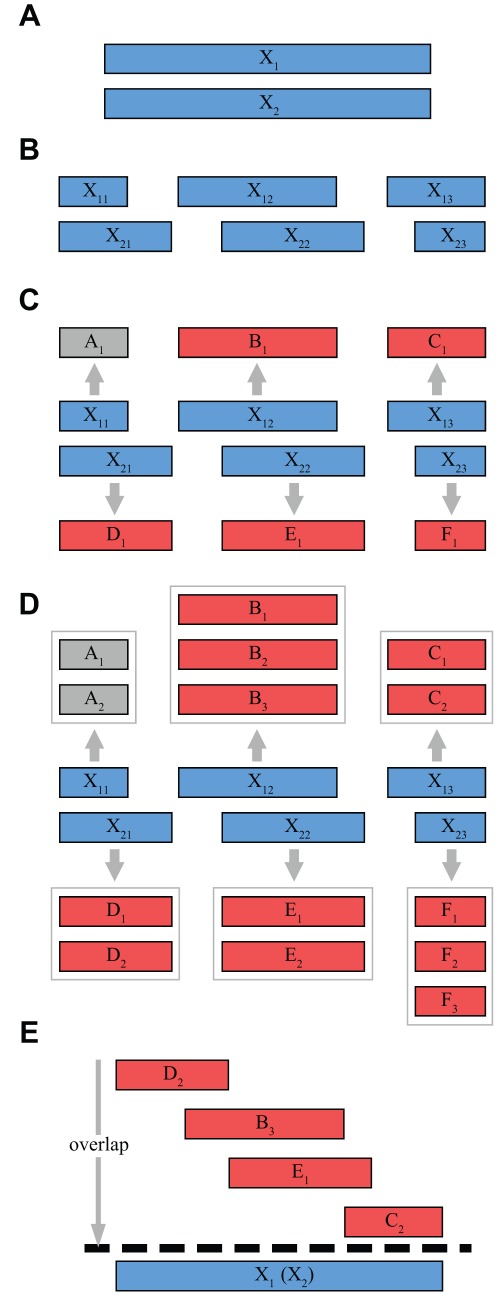
Schematic of the proposed sequencing method. The target sequence *X* is replicated multiple times (A) and the different replicas 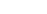 with 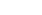 are randomly separated into fragments 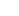 (B). Sequencing each fragment using classical SBH yields one possible ‘candidate’ 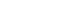 for each fragment’s sequence (C). Using the repeat structure of the candidate, the complete set of possible fragment sequences is constructed (D). Aligning the candidates allows to uniquely determine the target sequence and choose one element of each candidate set (E). The method is robust against erroneous candidate sets resulting from errors in the determined probe spectra of fragments, indicated as grey bars in (D).

Although a target sequence with a repeat structure that leads to multiple reconstructions cannot be uniquely identified with a single round of SBH, the set of target sequences compatible with the spectrum is finite. We now show that fragmenting multiple copies of a long target sequence and enumerating the complete sets of possible sequences for each fragment allows the unique identification of the target. Since all fragments have to be compatible with the same long target DNA, there is sufficient additional information to break the degeneracy, choose a specific fragment sequence and uniquely reconstruct a uniformly random target for 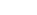. The proposed method is summarized in FIG. 2.

### Solution Enumeration

**Figure 3:**
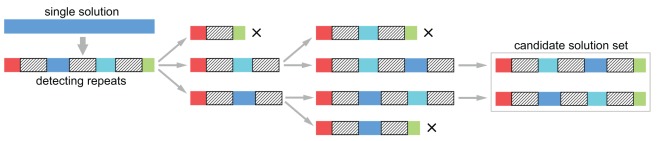
Constructing the complete candidate set. The algorithm first detects the locations of (l–1)-mer repeats in a single solution (leftmost column, repeats are shaded grey). Then we enumerate all possible extensions of the repeat (2nd column), continuing until we arrive at a set of candidate solutions that have the same length as the original sequence (3rd and 4th columns). Since all permutation of the subsequences between repeats are checked for consistency with the probe spectrum, all degenerate solutions are found.

To enumerate all possible fragment sequences consistent with a given probe spectrum, we first determine one solution using a standard reconstruction algorithm. FIG. 3 shows how the enumeration is done for a sequence containing a triple 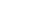-mer repeat. After detecting the locations of the repeats, the algorithm starts from the beginning of the known solution and enumerates all possible extensions after the first repeat. Continuing along the sequence, the process of enumerating all possible extensions and permuting parts of the sequence accordingly is iterated. The search is terminated when it reaches the end of the known solution, and we discard any search that does not cover the full length. Since an exhaustive search over all possible permutations of the 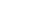-mer repeats is performed, the set of solutions is complete.

### Unique Reconstruction

Since all fragments stem from the *same* sequence, we can uniquely determine the target by choosing specific solutions for the individual fragments. In order to find a target sequence consistent with all the candidate sets we use a variant of ACO, which starts by randomly selecting the solution sets of some fragments. For each candidate in these sets, the algorithm iteratively determines its left or right successors by heuristically choosing a candidate from one of the remaining candidate sets. The optimum is the reconstruction of the target sequence.

## Discussion

### Testing the Method on Random Sequences

**Figure 4:**
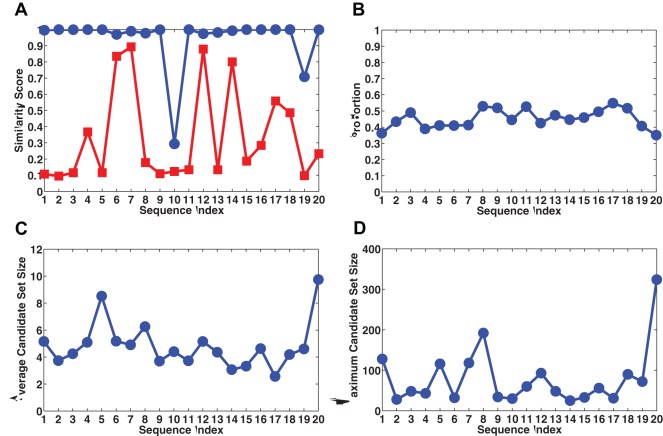
Performance of the method for random sequences. 20 randomly generated 5000-mers are sequenced using 7-mer probes assuming 5% positive and 5% negative random errors in the probe spectrum. Each target is replicated 8 times and separated into fragments of length randomly within 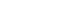. (A) Similarity score comparison between the proposed method (blue circles) and a control method (red squares). The control method does not generate complete sets of possible sequences for fragments, i.e. each candidate solution set contains exactly one candidate. (B) In contrast, due to reconstruction degeneracy and biochemical errors, the proportion of correctly reconstructed fragments is less than one half on average. (C), (D) The average and maximum number of candidates of fragments used in the assembly. The proposed method significantly outperforms the control method and allows to uniquely choose one out of up to 300 degenerate solutions of a fragment.

To test the method, we carry out simulations on 20 randomly generated 5000-mers, and attempt to sequence them using probes of length 7. We replicate each 5000-mer 8 times, randomly separate each replica into fragments of length 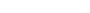, whose locations in the target are unknown, and include 5% positive and 5% negative errors into the spectrum of each fragment. Therefore, roughly 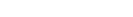 fragments need to be sequenced for each 5000-mer. Note that by choosing an average fragment length of 200 we are well above the traditional boundary for SBH, since 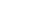. In the assembly, we begin with 8 randomly selected solution sets. FIG. 4A shows the performance comparison between the proposed method (blue circles) with a control method (red squares). The proposed method has an average similarity score of 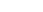 over the 20 trials, including 2 accidental drops which are presumably due to errors in the spectra, whereas the control method has an average similarity score of 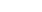. In contrast, FIG. 4B shows that only about half (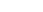 on average) of the fragments are correctly reconstructed. FIG. 4C and FIG. 4D illustrate the number of candidate solutions for fragments used in the assembly. The average number of solutions ranges from 2 to 10, whereas the maximum exceeds 300. Despite these degeneracies, the accuracy of the reconstruction of the entire 5000-mer is nearly perfect.

### Testing the Method on Natural Sequences

The repeat distribution of natural sequences is not random, and hence, when sequencing with probes of length *l*, the expected length of a fragment for non-unique solutions will be different than 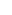. The present method will be useful when the fragment length is sufficiently long to have multiple interlacing repeats in the sequence, but not too long that prohibitively many replicas are needed to be analyzed to break the degeneracy. Unlike the case of random sequences, we do not know this length *a priori*, therefore we need to find it as part of the reconstruction procedure.

**Figure 5:**
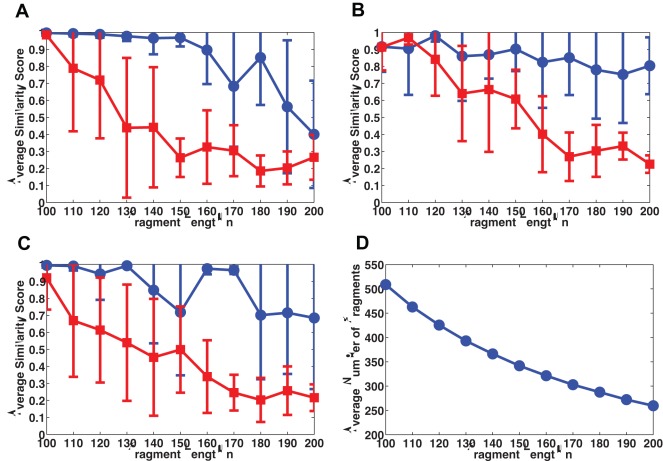
Performance of the method on natural sequences. The average similarity score over ten independent simulation runs on the 5000-mer prefix of three natural sequences (GenBank accession numbers JA638618 (A), AEQT01000438 (B) and AFZZ01000001 (C)), as a function of the fragment length *n* used to separate replicas. The results of both the proposed method (blue circles) and a control method (red squares) without generating a complete set of possible sequences for each fragment are shown. Error bars represent the standard deviation. Each 5000-mer is replicated 10 times and sequenced with 7-mers. 5% positive and 5% negative errors are included in the spectrum of each fragment. (D) shows the average number of fragments need to be sequenced as a function of the fragment length *n*.

**Figure 6:**
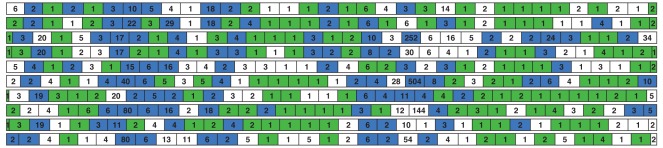
Visualization of replicas of the 5000-mer prefix of sequence JA638618 in one simulation attempt. Each row represents one replica of the sequence. Green bars represent fragments that are correctly reconstructed before solution enumeration, while blue bars represent fragments that are not correctly reconstructed, but the correct solutions appear in their candidate solution sets. The number of consistent solutions are shown in each fragment.

To verify this, we carry out simulations on the 5000-mer prefix of three natural sequences from human RNA and bacterial DNA respectively (GenBank accession numbers JA638618, AEQT01000438 and AFZZ01000001). In the absence of detailed information about the statistics of their repeats as a priori, we empirically change the fragment length *n* by drawing it randomly from intervals ranging from [90,110] to [190,210], i.e. from 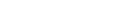 to 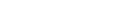, with step 10 Henceforth, we will use the center of the interval as a representative when describing fragment length. We replicate each target 10 times, and sequence them with 7-mers. 5% positive and 5% negative errors are added to the spectrum of each fragment. FIG. 5A, B and C respectively shows the average similarity score over ten independent reconstruction attempts, using both the proposed method (blue circles) and the control method (red squares), as a function of the fragment length for the three 5000-mers. FIG. 5D shows the average number of fragments need to be sequenced for different fragment length. With error bars representing the standard deviation, we can infer that the optimal fragment length for the sequences in FIG. 5 A and B are roughly 150 and 170, respectively, where sequencing 350 and 310 fragments give the proposed method larger than 95% in similarity scores, which significantly outperforms the control method. Note that the optimal fragment length increases when sequencing with more replicas. For the sequence in FIG. 5C, the optimal fragment length is unclear when sequencing with 10 replicas; however, we can still see the significant performance gain for the proposed method. Since different natural sequences have different optimal fragment length when separating replicas of the target, the optimal length of a target must be determined adaptively. For example, we could sequentially separate one replica, analyze its repeat pattern, and adaptively determine a better fragment length to separate the next replica. The computation time varies over sequences, since it depends on both the number and the aligning pattern of 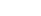-mer repeats in the target sequence at hand. For 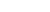, the average MATLAB computation time for the three 5000-mers are 1459 s, 690 s and 736 s respectively on a Mac Pro with two 2.26 GHz Quad-Core Intel Xeon processors.

The visualization in FIG. 6 further demonstrates the efficacy of our sequencing method. It illustrates one simulation attempt of the human RNA sequence (JA638618) when using the optimal fragment length (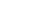) to separate replicas. Each row represents one replica of the 5000-mer. Although only 40.5% fragments are correctly reconstructed before solution enumeration (green bars), fragments that are not correctly reconstructed but have the correct solution in their candidate solution sets (blue bars) help to bridge gaps in the final assembly step, leading to near-perfect reconstruction.

To summarize, we have demonstrated how a variant of the classical Sequencing by Hybridization algorithm can significantly extend the length of target that can be sequenced with standard oligonucleotide probes. Our simulations indicate that both random and natural sequences of length 5000 can be accurately sequenced with standard 7-mer probes, even in the presence of 5% positive and negative errors.

## Supporting Information

Information S1 (PDF)
