# Supplementary material for: Sequencing by Hybridization of Long Targets
Source: PLoS One. 2012 May 4;7(5):e35819. doi: 10.1371/journal.pone.0035819 (PMC3344849; doi:10.1371/journal.pone.0035819)
Supplement: Information S1 — (PDF) [file pone.0035819.s001.pdf]

Supporting Information

Section A and B briefly introduce the algorithm to reconstruct short DNA fragment ($\sim$ 500 bases in length) from its spectrum. Most of what we cover here are replications of the work by Blazewicz et al. in [1] and Blum et al. in [2]. For more details, we recommend the original references to readers. Note that relevant studies are not restricted to them. [3] is a more general review. Section C describes the GUI we build to visualize the quality of reconstructions of benchmark instances.

A. Graph-Based Reconstruction

In our simulation, the reconstruction algorithm of short DNA fragments is based on a completely connected directed graph, which is an efficient strategy to handle both positive and negative errors. The method is proposed by Blazewicz et al. in [1]. For a simple example, suppose we sequence a target sequence

$$s_t = ACTGACTC$$

with probes of length 3, the ideal spectrum is

$$S_i = \{ACT, CTG, TGA, GAC, ACT, CTC\}.$$

Since in the biochemical experiment we can only read out a set of yes-or-no answers, we will miss the duplicated ACT. Besides, suppose there is a positive error TAA and a negative error CTG, then the output of the biochemical experiment will be the actual spectrum

$$S_a = \{ACT, TGA, GAC, CTC, TAA\}.$$

We can build a completely connected directed graph based on the actual spectrum, where vertices represent oligonucleotides in S_a, and the number of overlapping bases between oligonucleotides are weights of the directed edges, as FIG. 1(a) shows. The reconstruction of the target is equivalent to seeking the optimal path in the graph, which is to maximize $len(p)$ subject to

$$c(p) = l \times len(p) - \sum_{r=1}^{len(p)-1} o_{p(r)p(r+1)} \leq n,$$

where p is an arbitrary path in the graph; $p(r)$ is the rth vertex of p; $len(p)$ is the number of vertices in the path; n is the target sequence length; l is the probe length; o_{ij} is the number of overlapping bases from oligonucleotide i to j. Following a two-step procedure, we can algorithmically define the starting vertex of the path: first, find a set of vertices $S_{bs} \subseteq S_a$, such that the greatest-weighed outgoing edge of each vertex in S_{bs} is greater than or equal to the greatest-weighed outgoing edges of all other vertices in S_a; second, find a set $S_{wp} \subseteq S_{bs}$, such that the greatest-weighed incoming edge of each vertex in S_{wp} is less than or equal to the greatest-weighed incoming edges of all other vertices in S_{bs}. The vertex in S_{wp} (tie, if exists, is broken randomly) is defined as the starting vertex of the path. In FIG. 1(a), the path in red illustrates the optimal path of the graph. FIG. 1(b) shows the reconstruction of the target sequence by overlapping oligonucleotides in the optimal path.

B. Heuristic Algorithm

It has been proved that SBH with errors is strongly NP-hard [4]. Therefore, when the number of vertices becomes large, it is unpractical to find the solution to the above optimization problem by complete search. A number of heuristic algorithms has been developed. The simplest heuristics is the greedy algorithm, which always follows the outgoing greatest-weighed edge in each searching step after the first vertex is defined. However, this method is frail to biochemical error, and moreover, it will certainly encounter ambiguity at the searching step where the number of outgoing greatest-weighed edges is larger than one. A more elegant heuristics, which we adopt in our simulation, is the Ant Colony Optimization (ACO) algorithm [2].

The ACO imitates the mechanism of a swarm of ants searching for food. Ants deposit *pheromone* on the path they pass by to guide successive fellow ants. Eventually, the swarm of ants will converge to the shortest path between the net and the food source, since the amount of pheromone on this path grows to become the largest. In ACO, the algorithm searches path in the graph for many iterations, indexed by x. The edge from vertex i to j is not only weighed by their overlap o_{ij}, but also by a pheromone value $\tau_{ij}(x)$, which initially has the same value $\tau_{ij}(0)$ for $\forall i$, j but will keep updating in iterations according to a specified rule. The weighing function from i to j, μ_{ij}, is given by

$$\mu_{ij} = (\frac{o_{ij}}{l-1})^5 \tau_{ij}(x).$$

In each searching step, instead of using deterministic strategy, for a user-specified probability $q \in [0, 1)$, the next edge is chosen to be the greatest-weighed outgoing edge; otherwise the next edge is uniformly chosen from a candidate list, which contains a user-specified number of top-weighed outgoing edges. This nondeterministic strategy gives chance for the search to switch from greatest-weighed but incorrect edge to less-weighed but correct edge, and hence, the ACO is relatively robust to error and ambiguity. The final output is the path with the largest number of vertices, and tie, if exists, is broken by selecting the path with the most number of overlapping bases.

C. Visual Demonstration

We build a Java-based GUI [5], which shows the quality of reconstructions of natural DNA sequences taken from a benchmark library (The library that contains 10-mer repeats in [6]). The target sequence length is 509 while the probe length is 10. In the GUI, manipulating the slider on left switches the results of different instances of the benchmark. The first bar shows repetitive subsequences in the target sequence, where blocks with the same color represent the same repeat. Checking different check boxes under the bar shows either its 9-mer or 10-mer repeats. The next ten bars illustrate ten independent reconstructions of the target by ACO, with random 1% possitive and 1% negative errors added to each spectrum: the grey (pink) color represents bases that are correctly (incorrectly) matched by the reconstruction, with the Needleman-Wunsch similarity score [7] between the target and the reconstruction shown at the end of each bar. Over the entire benchmark, it appears that generally, the reconstruction failures are indeed correlated with multiple 9-mer repeats. Checking different check boxes at bottom right can switch between reconstruction from non-quantitative spectrum, obtained in practical experiment, and reconstruction from quantitative spectrum, which is an imaginary spectrum with repetitive oligonucleotides if the corresponding subsequence (10-mer) repeats itself in the target. It shows that adding quantitative information into the spectrum does not significantly improve the reconstruction.

[1] Blazewicz J, Formanowicz P, Kasprzak M, Markiewicz WT, Weglarz J (1999) DNA sequencing with positive and negative errors. *Journal of Computational Biology* 6:113–123.

[2] Blum C, Valles MY, Blesa MJ (2008) An ant colony optimization algorithm for DNA sequencing by hybridization. *Computers and Operations Research* 35:3620–3635.

[3] Xie H, Yuan Q, Liao L (2009) in *Bioinformatics and Biomedical Engineering, 2009. ICBBE 2009. 3rd International Conference on*, pp 1–4.

[4] Blazewicz J, Kasprzak M (2003) Complexity of DNA sequencing by hybridization. *Theoretical Computer Science* 290:1459–1473.

[5] http://people.seas.harvard.edu/∼qin/visrepeat/ (make sure the '∼' symbol is correctly input)

[6] Blazewicz J, Glover F, Kasprzak M (2005) Evolutionary approaches to DNA sequencing with errors. *Annals of Operations Research* 138:67–78.

[7] Needleman S, Wunsch C (1970) A general method applicable to the search for similarities in the amino acid sequence of two proteins. *Journal of Molecular Biology* 48:443–453.

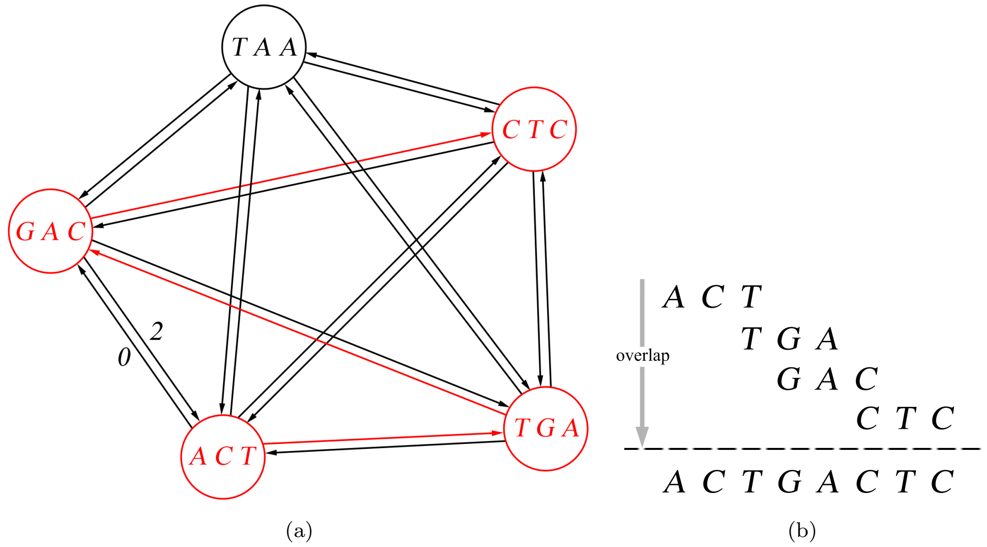

FIG. 1: **Graph-based reconstruction.** **(a)** The completely connected directed graph built from the spectrum $S_a = \{ACT, TGA, GAC, CTC, TAA\}$. Vertices represent oligonucleotides in S_a, and the number of overlapping bases between oligonucleotides are weights of the directed edges. For example, the weight of the edge from GAC to ACT is 2 since there are 2 overlapping bases, i.e. the AC; there is no overlapping base from ACT to GAC, therefore the corresponding weight is 0. Other weights are exempted from the figure for readability reason. The path in red illustrates the optimal path of the graph. **(b)** Reconstruct the target sequence by overlapping oligonucleotides in the optimal path.
